# Supplementing a Phytogenic Feed Additive Modulates the Risk of Subacute Rumen Acidosis, Rumen Fermentation and Systemic Inflammation in Cattle Fed Acidogenic Diets

**DOI:** 10.3390/ani12091201

**Published:** 2022-05-06

**Authors:** Raul Rivera-Chacon, Ezequias Castillo-Lopez, Sara Ricci, Renee M. Petri, Nicole Reisinger, Qendrim Zebeli

**Affiliations:** 1 Institute of Animal Nutrition and Functional Plant Compounds, University of Veterinary Medicine Vienna, Veterinärplatz 1, 1210 Vienna, Austria; raul.rivera-chacon@vetmeduni.ac.at (R.R.-C.); ezequias.castillo-lopez@vetmeduni.ac.at (E.C.-L.); sara.ricci@vetmeduni.ac.at (S.R.); renee.petri@agr.gc.ca (R.M.P.); 2 Christian Doppler Laboratory for Innovative Gut Health Concepts of Livestock, Veterinärplatz 1, 1210 Vienna, Austria; 3 Sherbrooke Research and Development Centre, Agriculture and Agri-Food Canada, 2000 College Street, Sherbrooke, QC J1M 0C8, Canada; 4 BIOMIN Research Center, BIOMIN Holding GmbH, 3430 Tulln, Austria; nicole.reisinger@dsm.com

**Keywords:** rumen pH, high concentrate, fermentation, cattle

## Abstract

**Simple Summary:**

The present study evaluated the hypothesis that phytogenic supplementation in the diet will reduce the negative impacts of subacute ruminal acidosis and modulate rumen fermentation. A control group of cows with no supplementation was compared to a group supplemented with 0.04% (DM basis) of a phytogenic feed additive. We observed that after the high-concentrate diet was implemented with the phytogenic blend based on L-menthol, thymol, eugenol, mint oil (*Mentha arvensis*) and cloves powder (*Syzygium aromaticum*), the mean ruminal pH increased and the time for pH to reach below 5.8 decreased during the last two weeks of the experiment. Phytogenic feed supplementation also increased ruminal acetate and butyrate and reduced propionate, promoting more stable rumen fermentation compared to no supplementation (Control). Acute phase proteins decreased with the phytogenic feed additive from week 3 of high concentrate feeding. Nevertheless, liver enzymes did not seem to be affected by supplementation. Our study demonstrated that acidogenic diets supplemented with a phytogenic compound can reduce the risk of subacute ruminal acidosis.

**Abstract:**

Feeding with high-concentrate diets increases the risk of subacute ruminal acidosis (SARA). This experiment was conducted to evaluate whether supplementing a phytogenic feed additive based on L-menthol, thymol, eugenol, mint oil (*Mentha arvensis*) and cloves powder (*Syzygium aromaticum*) (PHY) can amend the ruminal fermentation profile, modulate the risk of SARA and reduce inflammation in cattle. The experiment was designed as a crossover design with nine non-lactating Holstein cows, and was conducted in two experimental runs. In each run, cows were fed a 100% forage diet one week (wk 0), and were then transitioned stepwise over one week (0 to 65% concentrate, wk adapt.) to a high concentrate diet that was fed for 4 weeks. Animals were fed diets either with PHY or without (CON). The PHY group had an increased ruminal pH compared to CON, reduced time to pH < 5.8 in wk 3, which tended to decrease further in wk 4, reduced the ruminal concentration of D-lactate, and tended to decrease total lactate (wk 3). In wk 2, PHY increased acetate, butyrate, isobutyrate, isovalerate, and the acetate to propionate ratio compared to CON. Phytogenic supplementation reduced inflammation compared to CON in wk 3. Overall, PHY had beneficial effects on ruminal fermentation, reduced inflammation, and modulated the risk of SARA starting from wk 3 of supplementation.

## 1. Introduction

Feeding energy-dense diets with high levels of concentrates is necessary to meet the energy requirements and support the production performance of dairy cattle. High concentrate diets typically contain elevated levels of starch and low physically effective fiber (peNDF) [1]. Large amounts of starch are rapidly fermented into lactate and other short-chain fatty acids (SCFA) such as propionate, which is an important glucogenic precursor [2,3]. These events coupled with decreased salivary secretions as a result of low dietary peNDF may lead to decrease ruminal pH. Ruminal pH is crucial for the sustained activity of rumen microbiota [4]. A regular and intermittent reduction in ruminal pH, that typically starts around 4–8 h after the main meal of the day and lasts for several hours a day increases the risk of subacute ruminal acidosis (SARA) [5]. SARA often leads to systemic inflammation and increased odds for various health disorders in cattle [6]. The mechanisms behind SARA-induced systemic inflammation are not well understood, but it is believed that the drop in the pH combined with the release of microbial-derived toxins in the rumen increases the permeability of the rumen epithelium [7]. Once in the host bovine systemic circulation, microbial-derived toxins activate the proinflammatory cascade, leading to an increased secretion of inflammation markers such as serum amyloid A (SAA) and haptoglobin (Hp) [6,7].

Over the years, research has aimed to develop nutritive prevention strategies against SARA [4,5,8]. Yet, with diets containing more than 25% starch, as typically fed during lactation, SARA prevention is extremely difficult [1,9]. In such dietary conditions, the inclusion of various feed additives including phytochemicals have been shown to influence rumen fermentation, regulate ruminal pH and alter systemic metabolism [10,11]. Specifically, phytogenic additives have shown several benefits, including the potential to modulate rumination and enhance salivary secretions [12,13,14]. Furthermore, research conducted by our group when testing nine pure phytogenic compounds at two inclusion levels helped to positively influence salivary composition and ruminal fermentation profile [13,14]. However, despite those findings, most research has been limited to short-term effects, and the effects of a long-term supplementation in cows under a high concentrate (HC) challenge remains largely unknown. Therefore, the objectives of this study were to assess the effect of a phytogenic additive supplementation in cattle transitioned from a forage to HC diet and fed for four consecutive weeks on ruminal pH, SARA risk, SCFA profile, ammonia, lactate, and several biomarkers of inflammation and liver health. Our hypothesis states that supplementation with the phytogenic additive will mitigate the negative impacts of HC feeding by modulating ruminal pH and reduce lactate production, thus reducing the risk of SARA and associated systemic inflammation.

## 2. Materials and Methods

### 2.1. Animals, Experimental Design, Treatments and Management

The experiment used nine rumen-cannulated (Bar Diamond, Parma, ID, USA) non-lactating Holstein (992 ± 72.6 kg and 10 ± 0.8 years) cows in a cross-over experimental design with two experimental runs. The animals were group-housed at the research dairy farm of the University of Veterinary Medicine, Vienna (Pottenstein, Lower Austria). Cows were blocked in two groups with four and five animals based on body weight and randomly assigned to either a control diet (CON) with no supplementation or a diet supplemented with 0.04% (DM basis) of a phytogenic feed additive in powder form based on a blend of spices, extracts and herbs including L-menthol, thymol, eugenol, mint oil (*Mentha arvensis*) and cloves powder (*Syzygium aromaticum*) (PHY; Digestarom^®^, DSM GmbH, Grenzach-Wyhlen, Germany). Prior to the start of the experiment, the cows were adapted to the feeders with a forage-only diet for two weeks. Each experimental run consisted of six weeks. During the first week of each run (wk 0), the cows were fed a forage-only diet (F) including grass silage, corn silage and hay (Table 1). Then, the cows were transitioned step-wise during one week by increasing the proportion of concentrate in the total mixed ration (TMR) by 10% daily. The high concentrate (HC) TMR contained (DM basis) 26.25% grass silage, 8.75% corn silage, and 65% pelleted concentrate (Table 1), which was fed for four weeks. During wk 0 of forage feeding, the mineral and vitamin mix either with or without the phytogenic additive were dosed through the ruminal cannula of treatment cows. Throughout the adaptation (diet transition), the amount of supplementation dosed through the ruminal cannula was adjusted according to the level of dietary concentrate inclusion. Once cows consumed the high-concentrate diet, the mineral and vitamin mix was combined with the corresponding concentrate, and then integrated in the TMR. The level of inclusion of the PHY or CON supplementation was 0.04% of the TMR. The diets were formulated to meet or exceed the nutrient requirements of a dry cow (GfE, 2001). There was a washout period between experimental runs that lasted 4 weeks; during which, cows were fed only hay.

During the experiment, cows were housed in a free-stall barn with deep litter cubicles (2.6 × 1.25 m, straw litter) and free-choice mineral blocks. Water and feed was available for ad libitum consumption. The TMR was prepared once a day using an automatic feeding system (Trioliet Triomatic T15, Oldenzaal, The Netherlands), and offered in individual feeding troughs to the cows at approximately 0800 h. Individual feed intake was continuously controlled and recorded as feed bunks were equipped with electronic scales and computer-regulated access gates (Insentec B.V., Marknesse, The Netherlands) and checked daily before discarding the refusals. With the purpose of increasing palatability, and due to the low proportion of moisture of the TMR, water was added during mixing with a targeted DM content of approximately 46%. The ethical consent number of the present experiment was BMBWF-68.205/0003-V/3b/2019.

### 2.2. Feed Sampling and Chemical Analysis

The dry matter concentration of the TMR was determined every day by drying samples at 100 °C for 24 h. Individual feed samples were collected at the beginning and at the end of each experimental run, and samples of TMR were collected once a week for chemical composition. All nutrient analyses of feed samples were evaluated in duplicate according to the German Handbook of Agricultural Experimental and Analytical Methods (VDLUFA; Naumann and Bassler) [16]. The DM of wet feed samples was estimated by forced-air drying at 55 °C for 48 h and the residual water was subsequently analyzed by oven drying at 105 °C for 4 h (method 3.1). Ash was determined by combustion in a furnace oven at 580 °C overnight (method 8.1). Crude protein (CP) was estimated with the Kjeldahl method [16] and ether extracts (EE) using the soxhlet extraction system (Extraction System B-811, Büchi, Flawil, Switzerland). Similarly, for NDF and ADF (methods 6.5.1 and 6.5.2, respectively) concentrations were estimated with sodium sulfite and reported exclusive residual ash following the official analytical methods [16] using the Fiber Therm FT 12 (Gerhardt GmbH & Co. KG, Königswinter, Germany) with heat-stable α-amylase for NDF analysis. Starch content was measured with K-TSTA kit (Megazyme Ltd., Wicklow, Ireland). Non-fiber carbohydrates content was calculated as 100 − (% CP + % NDF + % EE + % ash). Acid detergent lignin was determined gravimetrically after ADF separation with 72% sulfuric acid.

Particle size distribution of the HC diets was determined according to Kononoff et al. [15] with a modified Penn State Particle Separator that included three screens (19.0, 8.0, and 1.18 mm) and a pan, with a minor adjustment. Briefly, because a certain portion of the concentrate pellets stayed on the 8 mm screen, after analysis for particle size distribution, an adjustment factor was applied to correct these values by hand picking the remaining pellets from the 8 mm screen and transferring them to the 1.18 mm screen. With this data, physically effective NDF (peNDF) and the physically effectiveness factor (pef) were calculated as outlined by Beauchemin and Yang [17]. The peNDF concentration of the diet was determined with the multiplication of NDF content of the diet by its pef. The pef (ranged from 0 to 1) was calculated as the sum of the proportion of particles retained on the corresponding sieves (19.0 and 8.0 mm sieves for pef > 8 mm).

### 2.3. Measurements of Ruminal pH and Monitoring of SARA 

Ruminal pH was continuously measured using the Lethbridge Research Centre Ruminal pH Measurement System (LRCpH; Dascor Inc., Oceanside, CA, USA) placed in the rumen ventral sac as outlined by Penner et al. [18]. Ruminal pH data were downloaded, and systems were calibrated every week using a pH 4 and 7 solution and were programmed to record pH every 15 min. To monitor the risk of SARA, we calculated minimum, mean and maximum ruminal pH, the difference between maximum and minimum pH, the time at which ruminal pH was below 5.8 and 6.0, and the area below ruminal pH 5.8 and 6.0, as SARA indicators [5,6]. Additionally, a SARA index was calculated using two approaches. First, by calculating the time that ruminal pH was below 5.8 per kg DMI, and then by calculating the area for which pH was below 5.8 per kg of DMI [19].

### 2.4. Collection of Ruminal and Reticular Fluid and Analysis

Approximately 10 mL of reticular fluid was collected manually through the rumen cannula with a disposable 20 mL syringe. After the rumino-reticular fold was reached and passed, the sample was aspirated from the ventral region of the reticulum. Ruminal fluid samples were collected from the ventral sac of the rumen. Samples were transferred to 15-mL vials and immediately frozen at −20 °C. At the end of experimental samplings, SCFA measurements were conducted following Qumar et al. [20] with minor modifications. Briefly, reticular and ruminal samples were thawed overnight at 4 °C, centrifuged at 3220× *g* for 20 min and the supernatant was used for further analysis. Then, 200 µL of distilled water, the internal standard 4-methylvaleric acid (Sigma-Aldrich, St. Louis, MO, USA) and 200 µL of 1.8 mol hydrochloric acid were added to 600 µL of supernatant. Samples were vortexed and then centrifuged at 20,000× *g* for 20 min at 4 °C. The clear supernatant was transferred into glass vials for the gas chromatograph. The analysis was conducted using a gas chromatography apparatus (Shimadzu GC Plus with FID detector) which was equipped with 30 m × 0.53 mm ID × 0.53 µm capillary column (Trace TR Wax, Thermo Fisher Scientific, Waltham, MA, USA). The injector and detector had temperatures of 170 °C and 220 °C, respectively. The gas used as carrier was Helium with a flow rate of 6 mL/min. Additionally, ruminal and reticular ammonia was determined using the indophenol reaction [21], and a lactate analysis was conducted following the Megazyme K-DATE assay (Megazyme Ltd., Wicklow, Ireland).

### 2.5. Blood Sampling and Analysis of Systemic Health Biomarkers

Blood samples were collected on a weekly basis before the morning feeding from the jugular vein; serum was obtained using 9-mL vacutainer tubes (Vacuette; Greiner Bio-One, Kremsmünster, Austria). Acute phase proteins concentration analyses including Hp and SAA were determined using a Tridelta phase range Multispecies SAA ELISA kit (Tridelta Development Ltd., Greystones, Co., Wicklow, Ireland), SAA serum samples were diluted 1:500 and samples with optical density values above the standard curve were diluted again (1:400 or 1:250) and analyzed once more. No dilution of serum was needed for Hp measurement. Liver enzymes including alkaline phosphatase (ALP), aspartate aminotransferase (AST), glutamate dehydrogenase (GLDH), and gamma-glutamyl transferase (GGT) were measured with a conventional large-scale analyzer for clinical chemistry at the laboratory of the Central Clinical Pathology Unit, University of Veterinary Medicine, Vienna. The standard enzymatic colorimetric analyses with a fully automated autoanalyzer for clinical chemistry (Cobas 6000/c501; Roche Diagnostics GmbH, Vienna, Austria) was used. The intra-assay variation was controlled by limiting the coefficient of variation to ≤10% for SAA and <5% for other blood variables.

### 2.6. Statistical Analysis

Statistical analyses were performed according to a crossover design [22] using the PROC MIXED of SAS (version 9.4; SAS Institute, Cary, NC, USA). Data were first checked for outliers using the Cook’s D with a 0.08 threshold used for outliers, which were removed from further analyses. The normality of data was evaluated with the PROC UNIVARIATE followed by the normal and plot options. If the normality assumption was not met, PROC TRANSREG performing a Box-Cox was used to determine the transformation mode, which was performed before the ANOVA. The statistical model included the fixed effects of run, diet, and treatment supplementation, as well as the interactions diet × week × treatment. The cow within the run was considered as a random effect, whereas data obtained from the same cow in different times were processed as repeated measures in the ANOVA, with a first order variance-covariance structure matrix, taking into consideration that the covariance decays with time. Data are reported as LSM and the transformed data were transformed back after the ANOVA. The largest standard error of the mean (SEM) is reported. Statistical significance was declared when *p* ≤ 0.05 and tendency is discussed if 0.05 < *p* ≤ 0.10. For a better visualization of the SCFA profile and fermentation pattern occurring in the rumen and reticulum, boxplot figures were constructed for data of ruminal and reticular individual SCFA with R [23] and using the ggplot2 package version 3.3.5 [24].

## 3. Results

### 3.1. Ruminal pH and SARA

Data showed a decreased ruminal pH after switching the diet from F to HC, and the ruminal pH depression was maintained throughout the experiment, as indicated by various SARA indicators measured (Table 2). However, in both wk 3 and 4 of HC feeding, the PHY supplementation reduced the risk of all SARA indices measured. In particular, supplementation increased mean and minimum ruminal pH compared to CON (*p* < 0.05). Furthermore, the supplemented cows showed a shorter time below 5.8 in both wk 3 and 4 of HC (148 vs. 287 min in wk 3, and 196 vs. 330 min in wk 4 for PHY and CON, respectively) and the area with pH < 5.8 tended to be lower for PHY compared to CON in wk 3 HC (*p* = 0.10). Consequently, the SARA index (time pH < 5.8/kg DMI) was lower for PHY compared to CON (*p* < 0.05) in wk 3 and 4 of HC feeding and the SARA index (area pH < 5.8/kg DMI) was lower for PHY compared to CON (*p* < 0.05) in wk 3 (Table 2). Additionally, PHY feed additive tended to decrease DMI compared to CON.

**Figure 1:**
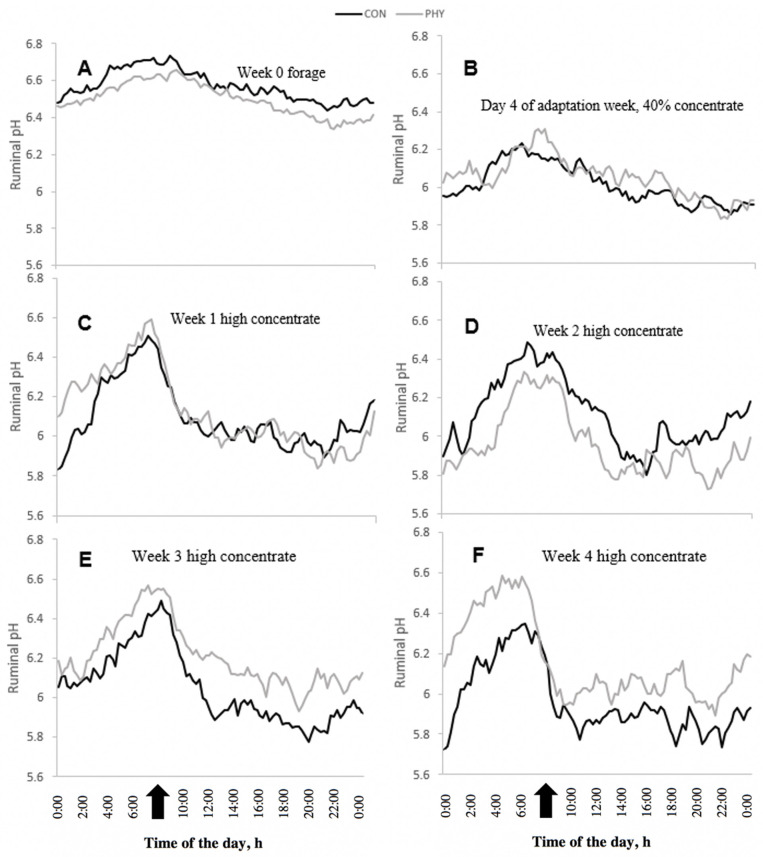
Diurnal variation of ruminal pH in various weeks in cows fed either forage-only or with 65% concentrate in a diet, without supplementation (CON) or supplemented (PHY) with a phytogenic feed additive based on L-menthol, thymol, eugenol, mint oil (*Mentha arvensis*) and cloves powder (*Syzygium aromaticum*). Time of feeding: 08:00 h (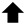). (**A**) Week 0 forage, SEM = 0.05. (**B**) Day 4 of adaptation week, 40% concentrate, SEM = 0.08. (**C**) Week 1 of high concentrate, SEM = 0.09. (**D**) Week 2 of high concentrate, SEM = 0.12. (**E**) Week 3 of high concentrate, SEM = 0.12. (**F**) Week 4 of high concentrate, SEM = 0.12. *p*-Values: Time < 0.01; Week < 0.01; Time × Week < 0.01. SEM: The largest standard error of the mean.

Daily ruminal pH oscillation was also impacted by diet composition, showing a larger variation for the HC compared to forage feeding (*p* < 0.01; Figure 1). During forage feeding, rumen pH oscillated between 6.72 and 6.34 at the time of feeding and 12 h later, respectively. During the fourth day of diet transition, ruminal pH ranged between 6.29 and 5.83 at 1 h prior and 13 h after feeding, respectively. The data showed that rumen pH peak was reached at the time of feeding whereas a low and relatively stable pH occurred 12 h after feeding. (Figure 1B–F). An improved response of ruminal pH in wk 3 and 4 of HC feeding was observed during the day (Figure 1E,F).

### 3.2. Ruminal and Reticular Short Chain Fatty Acids Profile

Data of SCFA profile indicated that there was an effect of the concentrate level (*p* < 0.01) on ruminal SCFA concentration (Table 3). Specifically, the total SCFA concentration increased by approximately 28% during HC feeding compared to wk 0 (forage feeding), with a maximum concentration of SCFA observed in wk 2 on HC diet consumption averaging 120.8 mM, independent of PHY supplementation (*p* = 0.85).

There was an increase (*p* < 0.01) in ruminal acetate, butyrate, isobutyrate, and isovalerate as well as a decrease (*p* < 0.01) in ruminal propionate with diet change. Interestingly, there was an interaction between diet, week of feeding and PHY supplementation on ruminal acetate, propionate, isobutyrate and isovalerate, with PHY supplementation displaying an increase in acetate from 52.6 to 55.4%, butyrate from 12.0 to 14.0%, isobutyrate from 0.80 to 1.0%, isovalerate from 1.43 to 1.90% and a decrease in propionate from 29.5 to 24.1% compared to CON in wk 2 of HC feeding. Moreover, PHY increased isovalerate from 1.65 to 2.08% in wk 3 compared to CON. Nevertheless, PHY increased the acetate to propionate ratio in wk 2 HC (*p* < 0.05) (Table 3).

**Figure 2:**
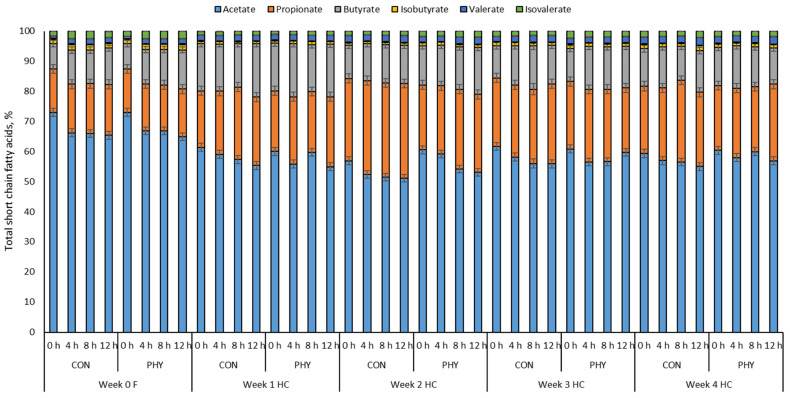
Variation of ruminal short chain fatty acid fermentation from 0 to 12 h post-feeding in cows fed either forage-only (F) or a high concentrate (HC), without supplementation (CON) or supplemented (PHY) with a phytogenic feed additive based on L-menthol, thymol, eugenol, mint oil (*Mentha arvensis*) and cloves powder (*Syzygium aromaticum*). *p*-Values: Acetate, Time < 0.01, Trt = 0.26, Diet < 0.01, Time × Trt × Diet × Week < 0.01; Propionate, Time < 0.01, Trt < 0.05, Diet < 0.01, Time × Trt × Diet × Week < 0.01; Butyrate, Time < 0.01, Trt = 0.23, Diet < 0.01, Time × Trt × Diet × Week < 0.01; Isobutyrate, Time = 0.36, Trt = 0.28, Diet < 0.01, Time × Trt × Diet × Week < 0.01; Valerate, Time < 0.01, Trt = 0.30, Diet < 0.01, Time × Trt × Diet × Week < 0.01; Isovalerate, Time = 0.45, Trt < 0.05, Diet < 0.01, Time × Trt × Diet × Week < 0.01.

Time post-feeding (*p* < 0.05) also showed an effect on total and individual proportions of SCFAs, with a reduction in acetate from 0 to 12 h after feeding, an increase in the proportion of propionate from 0 to 12 h post-feeding in wk 1 and 2 for both CON and PHY, wk 3 for CON and wk 4 for PHY. The proportion of butyrate increased with time post-feeding in wk 0 for both CON and PHY; this variable also increased post-feeding in wk 1 and 3 for CON and PHY, respectively (Figure 2).

In general, the fermentation and SCFA profile in the reticulum followed a pattern similar to that observed in the rumen (Figures S1 and S2). In the reticulum, PHY supplementation increased acetate, butyrate and isovalerate (*p* < 0.05) and decreased propionate (*p* < 0.05) compared to CON in wk 2 HC. Similarly, the A:P ratio increased with PHY compared to CON (*p* < 0.05) in wk 2 HC (Table S1). Influenced by time post-feeding (*p* < 0.05), acetate decreased consistently across weeks. However, propionate increased 12 h after feeding for both groups, and in wk 1 only for CON, whereas the proportion of butyrate increased after feeding in wk 0 and 3 for both CON and PHY, but this fatty acid displayed an increased post-feeding only in wk 2 for PHY (Figure S2).

### 3.3. Ruminal and Reticular Lactate and Ammonia

**Figure 3:**
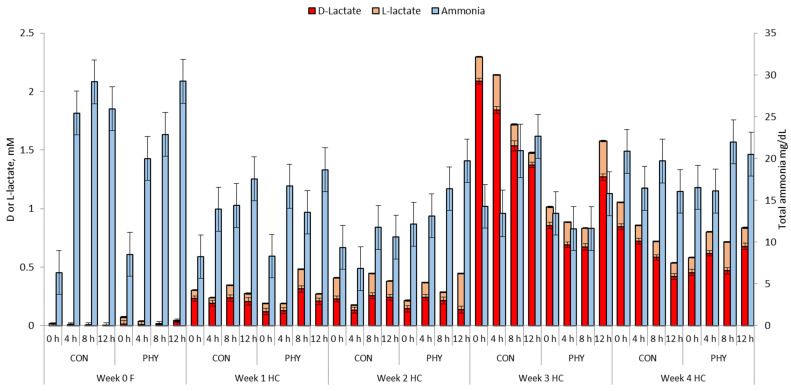
Variation of ruminal D-lactate, L-lactate (mM), and total ammonia concentration (mg/dL) from 0 to 12 h post-feeding in cows fed either all-forage (F) or a high concentrate (HC), without supplementation (CON) or supplemented (PHY) with a phytogenic feed additive based on L-menthol, thymol, eugenol, mint oil (*Mentha arvensis*) and cloves powder (*Syzygium aromaticum*). *p*-Values: D-lactate, Time = 0.99, Trt = 0.42, Diet < 0.01, Time × Trt × Diet × Week < 0.01; L-lactate, Time = 0.63, Trt = 0.60, Diet < 0.01, Time × Trt × Diet × Week < 0.05; Total ammonia, Time < 0.01, Trt = 0.90, Diet < 0.01, Time × Trt × Diet × Week < 0.01.

There was an increase in ruminal total lactate concentration (*p* < 0.05) when cows consumed the HC ration with average values of 0.17 mM and 1.10 for wk 0 and wk 4 HC, respectively (Table 3). An interaction between diet, week of feeding and PHY supplementation was observed for total and D-lactate concentration with a trend towards decreased total lactate (*p* = 0.06) and a reduction in D-lactate (*p* < 0.05) with PHY supplementation compared to CON in wk 3 HC. In addition, there was an interaction between diet, week of feeding and PHY supplementation for ruminal ammonia with this variable being greater for PHY cows in wk 2 HC (*p* < 0.05) compared to CON. Nevertheless, during wk 3 HC, PHY cows showed lower ruminal ammonia concentration (*p* < 0.05) compared to CON. Time of feeding did not influence total lactate throughout the experiment (Figure 3). However, in wk 3 HC at 0 h, PHY had a lower total lactate concentration (*p* < 0.05) and at 4 h after feeding PHY tended to reduce total lactate compared to CON (*p* = 0.06). Considering the influence of time of feeding, the total ammonia concentration in the rumen increased with increasing time post-feeding with an exception in wk 2, 4 HC CON, and wk 3, 4 HC PHY.

Total reticular lactate concentration showed a similar pattern to that observed in the rumen, with an overall increment from 0.32 mM (wk 0) to 1.16 mM (wk4). Similarly, PHY supplementation increased reticular ammonia concentration in wk 2 HC (*p* < 0.05), and during wk 3, HC decreased their reticular ammonia concentration compared to CON (*p* = 0.08) (Table S1). Total lactate concentration in reticulum was not influenced by time. However, that was not the case for L-lactate, which increased with time post-feeding, in wk 1 and 4 HC. Similarly, total ammonia increased after feeding, but not in wk 1, 2, 4 HC for CON and 3, 4 HC for PHY (Figure S3).

### 3.4. Systemic Inflammation and Liver Health Biomarkers

The SAA concentration increased almost 3-fold from forage feeding to HC feeding, whereas the Hp was not different (Table 4). Both APP were influenced by PHY supplementation after 2 weeks in feed. For example, PHY decreased the Hp blood concentration in wk 3 HC (*p* < 0.05) and tended to decrease it in wk 4 HC (*p* = 0.08), whereas PHY supplementation reduced SAA compared to CON in wk 3 HC (*p* < 0.05). The activity of liver enzymes AST, GLDH, and GGT were only influenced by changes in diet composition and increased with HC diet compared to wk 0 (*p* < 0.05); meanwhile, ALP did not change despite diet or supplementation (Table 4).

## 4. Discussion

In the present study, the HC diet contained an average of 28.5% starch and 18.0% peNDF > 8 mm, which, according to recent recommendations of Khorrami et al. [1], can be considered an acidogenic diet with a high risk of inducing SARA. Our main hypothesis stated that PHY supplementation will reduce the negative effects caused by SARA as the HC challenge is prolonged. In fact, we observed that from wk 3 HC, PHY started to modulate the pH and SARA indices, and this coincides with the decrease in D-lactate in the same week compared to CON. The risk of SARA has been largely characterized in the literature, with Zebeli et al. [5] suggesting a time threshold of 314 min per day with a pH below 5.8. This study also showed that a possible threshold of time when pH drops below 5.8 consistently during the 4 weeks of concentrate feeding could be 310 min per day, considering an average of the highest and lowest time below the SARA threshold and a 25% security margin. Kröger at al. [12] reported similar results, but with only two consecutive weeks of high concentrate after forage feeding and with a plant compound inclusion level of 3 g/cow/day. Other researchers [25] evaluating a mixture containing menthol also included levels from 3–6 g/cow/day. Phytogenic compounds such as menthol, levomenthol, β-linaloolm, anethole, hexadecanoic acid and ρ-menthane have been demonstrated to have similar effects and the potential for increasing rumen fluid pH in cows fed a 50:50 concentrate to forage ratio compared to a control diet [26]. Complementing this finding, Castillo-Lopez [13] reported that menthol tended to reduce the concentration of propionate in the rumen. This may have a positive effect on the consecutive weeks in terms of pH. For example, thymol, the main monoterpene phenol in thyme oil used in the PHY blend, contains p-cymene and γ-terpinene [27], which have demonstrated a positive influence in terms of modulating rumen pH, as well as menthol [28], the main component of essential oils of peppermint. Similarly, eugenol tended and increased ruminal pH in high grain diets [29,30], respectively; suggesting a positive effect on rumen microbial population [31,32]. Therefore, we speculate that the mechanisms by which these phytogenic compounds modulate ruminal fermentation may be through the action of the ruminal microbial community, particularly the acetate- and propionate-producing bacteria, which are largely responsible of fermentation and acid production in the rumen. However, the microbial community was not evaluated in the present study; thus, we are unsure which microbial taxa were affected.

In this study, CON cows experienced severe SARA, as indicated by increased time spent with a pH below 5.8, compared to PHY in wk 3 and 4 of HC. A plausible explanation for the higher mean ruminal pH and decreased SARA indices observed with PHY supplementation in wk 3 of HC feeding is a slower rate of starch degradability in the rumen [33]. This is supported by the lower D-lactate concentration indicating a decreased production of lactate. Our results suggest a positive impact of PHY in mitigating the detrimental effects of high concentrate feeding and decreasing the risk for SARA by rumen fermentation in high concentrate diets. Additionally, D-lactate increased from wk 1 and 2 compared to wk 3 by 2.5 and 2.3 fold for CON and PHY, respectively. The low increment of D-lactate in PHY further supports the modulation of rumen fermentation with supplementation. Our results coincide with Qumar et al. [20], as ruminal lactate displayed approximately a 10-fold increment in their study when cows transitioned to a high grain ration. Other authors have reported that cattle experiencing SARA showed concentrations of lactate of 2.29 mM [34] with high-yield lactating cows. In our experiment, ruminal lactate reached maximum values of 1.54 and 1.18 mM in wk 3 of HC for CON and PHY, respectively. To the best of our knowledge, there are no reports showing the impact of supplementing phytogenic compounds on lactate fermentation. However, discrepancies with our results, and those reported in the literature for lactate during SARA may be based on the method of detection, physiological stage of cows, and values below the detection limit. A possible explanation for the tendency to decrease DMI goes together with a more stable change in daily DMI as demonstrated by Stone [8] indicating that abrupt changes in intake can be reflected in stronger dietary SARA insults.

Our results showed that, in general, the fermentation pattern and SCFA profile between the reticulum and the rumen were similar. This reflects the close connection and constant exchange of digesta between compartments, with the difference that the first one is constantly buffered with saliva and carbon dioxide removal. Minor differences in lactate or ruminal SCFA fatty acid concentration noted between the rumen and the reticulum may be explained by differences in the physiology of both compartments. Differences in the water and saliva influx into the reticulum may distinguish it from the rumen and could influence the SCFA profile. High concentrate diets have also been shown to decrease ruminal acetate, isobutyrate and the acetate to propionate ratio, and to increase propionate and valerate in rumen fluid for two [11] or four consecutive weeks on high concentrate [35]. In addition, the accumulation of ruminal SCFA increases the risks for SARA. Thus, during the last few years, there has been a special interest in the use of essential oils, phytogenic additives and secondary plant compounds in ruminant diets to positively modulate rumen fermentation and to improve its efficiency [10]. Supplementation with phytogenic compounds has been shown to result in lower propionate concentrations in the rumen in a diet with a 50:50 concentrate to forage ratio [25]. In addition, Neubauer et al. [11] reported an increase in ruminal butyrate in high concentrate feeding after supplementing with phytogenic compounds in the TMR, and those results are in agreement with our findings in wk 2 of HC. 

The variation in the proportions of individual SCFA in the rumen can be influenced by diet composition or the rate of production and utilization of those acids. For example, the reduction in acetate and the increase in propionate with time post-feeding found in the present experiment, especially in wk 4 of HC, may be explained by the increased availability of readily fermentable carbohydrates in the HC diet. Interestingly, we found that in wk 2 propionate was consistently lower for PHY compared to CON post-feeding, which indicates the regulation of activity of propionate-producing bacteria with PHY supplementation immediately after diet consumption and up to 12 h post-feeding. Other reports have found that eugenol did not modify SCFA fermentation when included in diets of lactating cows with a 47:53 concentrate to forage ratio mainly including corn and soybean meal with different inclusion levels (25–75 mg/kg DM) [36]. Differences in the effect of phytogenic compounds on ruminal fermentation and function may also be explained by differences in the composition or dosages used, as well as rumen microbial activity or absorption rate.

Ruminal ammonia concentration followed an opposite pattern to that observed for SCFA and lactate as reported by Sato [37]. Specifically, the decrease in ruminal pH was associated with a decrease in ammonia concentration. Lana et al. [38] demonstrated that better assimilation of ammonia is not only influenced by increased microbial synthesis in high concentrate diets but also by the deamination rate. These results possibly reflect a more efficient microbial use of ammonia during high grain diet [39]. Another possible explanation may be related to changes in the abundance of hyper-ammonia producing bacteria closely involved in the generation of ammonia in the rumen due to phytogenic compounds [40], which would influence the concentration of ammonia, as observed in wk 2 and 3 of HC. However, it has been reported that feed additives did not show an effect on ammonia concentration after feeding a high concentrate diet (65% DM) for two consecutive weeks [11]. Discrepancies between our results and those from Neubauer et al. [11] may be explained by the shorter length of the HC challenge. In this study, the peak of total ammonia concentration was reached mostly 8 or 12 h after feeding similar than results reported by Sato [37] with rumen ammonia peak at 8 h after feeding in beef cattle.

The HC feeding and SARA was associated with increased inflammation markers, especially SAA. In this study, the SAA increased starting with wk 1 HC and this elevated APR was maintained throughout the trial for 4 weeks, being more pronounced in the CON group. However, preventive supplementation in 3 weeks demonstrated a decreased inflammation response (wk 3 and 4) compared to CON. Thus, PHY seemed to reduce the release and/or transfer of microbial toxins and biogenic amines to the bloodstream or to ease inflammation by increasing the hepatic clearance via bile [7,41], regulating the APR and inflammation in cattle, as reported by Yang et al. [42] and Oh et al. [43]. Rodrigues et al. [44] found that a mixture of condensed tannins fed to a group of lactating cows when fed a diet with 39% concentrate diet had a lower Hp concentration than the control group which is similar to our experiment during wk 3 and 4 HC. Interestingly, Yang et al. [42] reported a reduction in SAA when steers were supplemented with plant compounds similar to our results for wk 3 HC. Furthermore, liver enzymes increase when there is damage present in the tissue and, as a result, new organelles are produced to counteract inflammation. In the present study, the threshold values listed by Wille et al. [45] for activities of GLDH, and GGT (10.5 and 27 U/L, respectively) were exceeded from wk 3 of HC, suggesting a negative impact of high grain feeding on animal systemic health, and an impairment of liver function. The increase in the activity of GLDH and GGT after the change from a forage to HC diet coincided with results reported by Kröger et al. [46] once lactating cows were switched from a ration with 40:60 concentrate to forage ratio to one with 60% concentrate and may be explained by liver damage [47]. Furthermore, Lakhani et al. [48] reported similar results for ALP, but an increase in AST after the inclusion of a phytogenic feed additive in buffalos fed a 50:50 concentrate forage ratio. However, those results contrast findings from the present study, where systemic GGT did not change with PHY supplementation. Other reports have suggested that plant compounds can suppress oxidative stress and improve liver health status in different animal species [49]. Finally, our results may also suggest that animals in other physiological conditions such as non-lactating cows with a reduced DMI and nutrient demands show different results to the reports in the literature.

## 5. Conclusions

Overall, the study showed that a 65% concentrate diet successfully induced SARA and that supplementation with a phytogenic feed additive during the HC diet improved rumen SCFA profiles, and increased rumen pH after 2 weeks of high concentrate feeding. The improved rumen fermentation, reduced SARA indices and decreased inflammation response suggest a supplementation strategy of at least three weeks for PHY to contribute to reduce the negative effects of high concentrate feeding in dairy cattle. The PHY blend of L-menthol, thymol, eugenol, mint oil (*Mentha arvensis*) and cloves powder (*Syzygium aromaticum*) possibly influenced the microbial population by modulating rumen fermentation. Therefore, supplementation with 0.04% of the phytogenic compound mixed in the TMR can be recommended to lower the impact of high-concentrate feeding in dairy cows.

## Figures and Tables

**Table 1:** Ingredients, chemical composition, particle size distribution of diets fed to cows during the week of forage feeding and the high concentrate feeding period.

Item	Diet and Treatment ^1^		
	Forage Diet	High Concentrate Diet	
		CON	PHY
Ingredients (% of DM)			
Grass silage	75	26.25	26.25
Corn silage	15	8.75	8.75
Grass hay	10	0	0
CONTROL concentrate ^1^	0 *	65	0
TREATMENT concentrate ^2^	0 *	0	65
Chemical composition (% of DM unless stated)			
DM, % as fresh	32.4 ± 5.16	45.1 ± 0.83	44.0 ± 2.09
Crude protein (CP)	17.2 ± 1.08	19.6 ± 0.80	19.3 ± 1.15
Neutral detergent fiber (NDF)	50.4 ± 1.58	30.2 ± 2.09	31.6 ± 2.44
Acid detergent fiber (ADF)	36.6 ± 6.39	19.9 ± 2.12	20.0 ± 1.89
Starch	4.2 ± 1.3	28.9 ± 1.85	28.0 ± 1.59
Ether extract (EE)	2.9 ± 1.32	3.2 ± 0.16	3.2 ± 0.21
Non-fiber carbohydrates	18.4 ± 0.47	39.5 ± 1.85	39.0 ± 1.83
Ash	11.0 ± 1.87	6.8 ± 0.26	6.7 ± 0.25
Particle fraction (% retained) ^3^			
Long	86.7	27.8 ± 4.95	29.2 ± 6.57
Medium	5.54	29.3 ± 1.74	29.7 ± 2.55
Short	7.30	20.3 ± 2.20	18.8 ± 3.21
Fine	0.50	1.4 ± 0.93	1.1 ± 0.80
pef ^4^ _> 8 mm_	0.92	0.6 ± 0.02	0.6 ± 0.04
peNDF ^5^ _> 8 mm,_ % of DM	47.5	17.3 ± 0.71	18.6 ± 0.25

^1^ CON: The CON pelleted concentrate mixture contained: barley (30.22%), triticale (18.1%), bakery by-product (23.08%), rapeseed meal (24.0%), molasses (3.0%), mineral-vitamin premix for dairy cattle (0.8%), limestone (0.5%), and salt (0.3%); ^2^ PHY: The pelleted PHY concentrate mixture contained: barley (30.22%), triticale (18.04%), bakery by-product (23.08%), rapeseed meal (24.0%), molasses (3.0%), mineral-vitamin premix for dairy cattle (0.8%), limestone (0.5%), and salt (0.3%). In addition, it was formulated to provide 0.04% of a phytogenic feed additive based on L-menthol, thymol, eugenol, mint oil (*Mentha arvensis*) and cloves powder (*Syzygium aromaticum*) in the TMR; * 100 g of mineral and vitamin mix (16% Ca, 8% P, 11.5% Mg, 2.2% Na, 16.2 g Mn, 24 g Zn, 3.6 g Cu, 0.27 g Co, 0.54 g I, 0.13 g Se, 2300 kIU Vit A, 240 kIU Vit D, 5 g Vit E, 2 g Vit B1 per kg feed) without (CON) or with a phytogenic feed additive based on L-menthol, thymol, eugenol, mint oil (*Mentha arvensis*) and cloves powder (*Syzygium aromaticum*) (PHY) was added in the rumen through the ruminal cannula before morning feeding; ^3^ Particle fractions determined by Penn State Particle Separator with a 19 mm screen (long), 8 mm screen (medium), 1.18 mm screen (short), and a pan (fine) according to Kononoff et al. [15]; ^4^ Physical effectiveness factor; ^5^ Physically effective NDF.

**Table 2:** Effect of supplementation with a phytogenic feed additive based on L-menthol, thymol, eugenol, mint oil (*Mentha arvensis*) and cloves powder (*Syzygium aromaticum*) on ruminal pH parameters in cows consuming a forage diet or a high concentrate diet ^1^.

Item	Forage Diet Week 0		High Concentrate Week 1		High Concentrate Week 2		High Concentrate Week 3		High Concentrate Week 4			*p*-Values ^3^		
	CON	PHY	CON	PHY	CON	PHY	CON	PHY	CON	PHY	SEM ^2^	D	T	I
DMI, kg	8.43	7.57	13.31	12.85	13.93	13.09	14.09	13.32	12.91	11.77	0.602	<0.01	0.08	0.23
Maximum pH	6.89	6.82	6.64	6.60	6.71	6.66	6.60	6.64	6.59	6.66	6.7 × 10^−11^	<0.01	0.76	0.10
Minimum pH	6.30	6.31	5.61	5.60	5.50	5.44	5.52 ^b^	5.66 ^a^	5.51 ^b^	5.67 ^a^	9.2 × 10^−10^	<0.01	0.12	<0.01
Mean pH	6.58	6.56	6.04	6.05	6.03	6.00	6.02 ^b^	6.15 ^a^	6.02 ^b^	6.16 ^a^	2.8 × 10^−10^	<0.01	0.17	<0.05
Difference *	0.55 ^a^	0.48 ^b^	1.01	0.99	1.22	1.22	1.05	0.96	1.07	0.99	0.042	<0.01	<0.05	<0.01
Dur ^4^ 6.0, min *	2.45	0.31	581.2	651.9	620.5	662.4	538.3 ^a^	364.2 ^b^	653.1 ^a^	410.9 ^b^	2.12	<0.01	<0.05	<0.01
Dur ^4^ 5.8, min *	1.27	0.62	239.6	244.2	304.7	349.1	286.8 ^a^	148.4 ^b^	330.1 ^x^	195.5 ^y^	2.64	<0.01	0.07	<0.05
Area 6.0, min × pH *	1.13	0.01	143.6	115.6	169.0	184.3	108.3 ^a^	49.9 ^b^	146.9 ^a^	69.67 ^b^	1.48	<0.01	<0.05	<0.01
Area 5.8, min × pH *	0.20	0.00	68.06	38.89	92.14	89.48	51.87 ^x^	23.80 ^y^	70.85	42.72	1.29	<0.01	<0.05	<0.05
Acidosis index, area pH < 5.8/kg DMI *	0.01	0.00	5.16	3.18	7.21	7.44	4.75 ^a^	1.75 ^b^	6.99	3.08	0.16	<0.01	<0.05	<0.05
Acidosis index, time pH < 5.8/kg DMI *	0.09	0.02	17.88	18.60	22.82	27.00	20.92 ^a^	10.83 ^b^	30.93 ^a^	13.20 ^b^	0.25	<0.01	<0.05	<0.01

^1^ CON: A control diet containing no phytogenic product; PHY: supplementation with a phytogenic feed additive based on L-menthol, thymol, eugenol, mint oil (*Mentha arvensis*) and cloves powder (*Syzygium aromaticum*); ^2^ The largest standard error of the mean; ^3^ *p*-Values for the effect of diet (D), phytogenic treatment (T) and the diet × week × treatment interaction (I); ^4^ Duration (weeks of high concentrate feeding); * Values were transformed using the root square function after checking for normal distribution, and were transformed back after the analysis; ^a,b^ Means with different superscripts indicate a significant difference (*p* < 0.05) between CON and PHY; ^x,y^ Means with different superscripts indicate a tendency for significant differences (0.05 < *p* ≤ 0.10) between CON and PHY.

**Table 3:** Effect of supplementation with a phytogenic feed additive based on L-menthol, thymol, eugenol, mint oil (*Mentha arvensis*) and cloves powder (*Syzygium aromaticum*) on ruminal short chain fatty acid profile, ammonia and lactate in cows consuming a forage diet or a high concentrate diet ^1^.

Item	Forage Diet Week 0		High Concentrate Week 1		High Concentrate Week 2		High Concentrate Week 3		High Concentrate Week 4			*p*-Values ^3^		
	CON	PHY	CON	PHY	CON	PHY	CON	PHY	CON	PHY	SEM ^2^	D	T	I
Total SCFA concentration, mM	86.0	84.0	105	108	116	118	106	98.0	112	114	4.98	<0.01	0.82	0.71
% of total SCFA														
Acetate	67.4	67.8	58.1	56.9	52.6 ^b^	55.4 ^a^	57.8	57.1	56.9	57.9	0.76	<0.01	0.57	<0.01
Propionate	15.6	15.1	20.5	21.6	29.5 ^a^	24.1 ^b^	23.9	23.3	23.7	23.2	0.70	<0.01	0.08	<0.01
Butyrate	10.5	10.8	16.0	16.7	12.0 ^b^	14.0 ^a^	12.6	12.9	12.8	12.8	0.60	<0.01	0.16	0.17
Isobutyrate	1.70	1.80	0.84	0.90	0.80 ^b^	1.00 ^a^	1.15	1.18	1.23	1.11	0.07	<0.01	0.49	0.01
Isovalerate	2.17	2.29	1.27	1.30	1.43 ^b^	1.90 ^a^	1.65 ^b^	2.08 ^a^	1.92	1.78	0.08	<0.01	<0.05	<0.01
Valerate	1.48	1.45	1.90	1.98	2.22	2.19	2.01	2.06	2.29	2.37	0.08	<0.01	0.70	0.87
Ratio of acetate to propionate	4.39	4.54	2.86	2.77	1.90 ^b^	2.60 ^a^	2.46	2.67	2.37	2.68	0.15	<0.01	0.20	<0.01
Ammonia, mg/dL	21.72	20.16	13.53	14.30	9.64 ^b^	15.35 ^a^	17.66 ^a^	13.12 ^b^	18.26	18.77	1.61	<0.01	0.88	<0.01
Lactate ^4^														
D-lactate, mM	0.037 ^b^	0.062 ^a^	0.433	0.347	0.386	0.317	1.053 ^a^	0.765 ^b^	0.718	0.669	0.0027	<0.01	0.35	<0.01
L-lactate, mM	0.062	0.131	0.259	0.260	0.293	0.231	0.372	0.408	0.363	0.386	0.0010	<0.01	0.29	<0.01
Total lactate, mM	0.118	0.218	0.711	0.618	0.714	0.558	1.539 ^x^	1.183 ^y^	1.107	1.089	0.0033	<0.01	0.46	<0.01

^1^ CON: A control diet containing no phytogenic product; PHY: supplementation with a phytogenic feed additive based on L-menthol, thymol, eugenol, mint oil (*Mentha arvensis*) and cloves powder (*Syzygium aromaticum*); ^2^ The largest standard error of the mean; ^3^ *p*-Values for the effect of diet (D), phytogenic treatment (T) and the diet × week × treatment interaction (I); ^4^ Values were transformed using the root square function after checking for normal distribution, and were transformed back after the analysis; ^a,b^ Means with different superscripts indicate a significant difference (*p* < 0.05) between CON and PHY; ^x,y^ Means with different superscripts indicate a tendency for significant differences (0.05 < *p* ≤ 0.10) between CON and PHY.

**Table 4:** Effect of supplementation with a phytogenic feed additive based on L-menthol, thymol, eugenol, mint oil (*Mentha arvensis*) and cloves powder (*Syzygium aromaticum*) on liver enzymes and acute phase proteins in cows consuming a forage diet or a high concentrate diet ^1^.

Item ^4^	Forage Diet Week 0		High Concentrate Week 1		High Concentrate Week 2		High Concentrate Week 3		High Concentrate Week 4			*p*-Values ^3^		
	CON	PHY	CON	PHY	CON	PHY	CON	PHY	CON	PHY	SEM ^2^	D	T	I
Hp, μg/mL	103.0	141.9	142.3	308.6	257.9	452.0	621.2 ^a^	90.6 ^b^	429.4 ^x^	96.4 ^y^	1.82	0.12	0.46	0.19
SAA, μg/mL	2.83	2.86	10.25	15.04	19.61	29.85	28.67 ^a^	8.87 ^b^	12.7	9.14	1.53	<0.01	0.60	0.23
ALP, U/L	7.70	7.10	7.43	8.10	7.59	8.33	8.49	8.20	6.78	7.33	1.10	0.50	0.73	0.27
AST, U/L	67.85	72.80	63.21	66.40	76.28	86.98	98.06	94.92	85.31	98.12	1.08	<0.01	0.32	<0.01
GLDH, U/L	4.80	6.01	5.56	5.22	9.94	10.87	13.26	12.17	10.86	11.67	1.20	<0.01	0.78	<0.01
GGT, U/L	21.21	20.99	21.56	20.89	25.64	25.11	30.78	25.64	29.26	29.59	1.09	<0.01	0.57	<0.01

^1^ CON: A control diet containing no phytogenic product; PHY: supplementation with a phytogenic feed additive based on L-menthol, thymol, eugenol, mint oil (*Mentha arvensis*) and cloves powder (*Syzygium aromaticum*); ^2^ The largest standard error of the mean; ^3^ *p*-Values for the effect of diet (D), phytogenic treatment (T) and the diet × week × treatment interaction (I); ^4^ Values were transformed using the log function after checking for normal distribution. Hp: Haptoglobin, SAA: Serum Amyloid A, ALP: Alkaline Phosphatase, AST: Aspartate aminotransferase, GLDH: Glutamate Dehydrogenase, GGT: Gamma-Glutamyl Transferase; ^a,b^ Means with different superscripts indicate a significant difference (*p* < 0.05) between CON and PHY; ^x,y^ Means with different superscripts indicate a tendency for significant differences (0.05 < *p* ≤ 0.10) between CON and PHY.

## Data Availability

Not applicable.

## Supplementary Materials

The following supporting information can be downloaded at: https://www.mdpi.com/article/10.3390/ani12091201/s1, Table S1: Effect of supplementation with a phytogenic feed additive on reticular short chain fatty acid profile, ammonia and lactate in cows consuming a forage diet or a high concentrate diet ^1^; Figure S1: Boxplots illustrating the fermentation pattern and short chain fatty acid profile in the reticulum and rumen with time post-feeding according to diet and duration of high concentrate feeding in Holstein cows without supplementation (CON) or supplemented with a phytogenic feed additive (PHY) based on L-menthol, thymol, eugenol, mint oil (*Mentha arvensis*) and cloves powder (*Syzygium aromaticum*); Figure S2: Variation of reticular short chain fatty acid fermentation from 0 to 12 h post-feeding in cows fed either all-forage (F) or a high concentrate (HC), without supplementation (CON) or supplemented with a phytogenic feed additive (PHY) based on L-menthol, thymol, eugenol, mint oil (*Mentha arvensis*) and cloves powder (*Syzygium aromaticum*); Figure S3: Variation of reticular D-lactate, L-lactate (mM), and total ammonia concentration (mg/dL) from 0 to 12 h post-feeding in cows fed either all-forage (F) or a high concentrate (HC), without supplementation (CON) or supplemented with a phytogenic feed additive (PHY) based on L-menthol, thymol, eugenol, mint oil (*Mentha arvensis*) and cloves powder (*Syzygium aromaticum*).
