# Supplementary material for: Supplementing a Phytogenic Feed Additive Modulates the Risk of Subacute Rumen Acidosis, Rumen Fermentation and Systemic Inflammation in Cattle Fed Acidogenic Diets
Source: Animals (Basel). 2022 May 6;12(9):1201. doi: 10.3390/ani12091201 (PMC9105827; doi:10.3390/ani12091201)
Supplement: Supplementary file 1 [file animals-12-01201-s001.zip › animals-1670296-supplementary.pdf]

Table S1. Effect of supplementation with a phytogenic feed additive based on L-menthol, thymol, eugenol, mint oil (*Mentha arvensis*) and cloves powder (*Syzygium aromaticum*) on reticular short chain fatty acid profile, ammonia and lactate in cows consuming a forage diet or a high concentrate diet [1].

	**Forage Diet Week 0**		**High Concentrate Week 1**		**High Concentrate Week 2**		**High Concentrate Week 3**		**High Concentrate Week 4**			***p*-Values[3]**		
**Item**	**CON**	**PHY**	**CON**	**PHY**	**CON**	**PHY**	CON	PHY	CON	PHY	SEM[2]	D	T	I
Total SCFA concentration, mM	74.1	77.2	105	96.2	107	101	109[x]	94.0[y]	108	109	3.36	<0.01	0.04	0.09
% of total SCFA														
Acetate	66.4	66.6	58.1	57.1	52.5 [b]	55.8 [a]	58.3	58.0	58.0	58.4	0.75	<0.01	0.56	<0.01
Propionate	16.2	15.8	20.8	21.8	29.6 [a]	23.8 [b]	23.5	22.8	23.5	23.2	0.71	<0.01	0.12	<0.01
Butyrate	10.4	10.6	15.6	16.1	12.0 [b]	14.0 [a]	12.7	13.0	12.7	12.7	0.53	<0.01	0.21	0.06
Isobutyrate	1.89	1.94	0.95	1.03	0.88	1.08	1.15	1.23	1.14	1.07	0.06	<0.01	0.40	<0.01
Isovalerate	2.40	2.53	1.40	1.36	1.54 [b]	1.98 [a]	1.66 [y]	1.97 [x]	1.62	1.57	0.11	<0.01	0.31	<0.01
Valerate	1.72	1.75	2.09	2.14	2.43	2.36	2.03	2.04	2.11	2.28	0.07	<0.01	0.55	0.63
Ratio of acetate to propionate	4.07	4.21	2.78	2.62	1.76 [b]	2.33[a]	2.47	2.53	2.45	2.51	0.10	<0.01	0.19	<0.01
Ammonia, mg/dL	19.11	20.01	12.52	13.91	10.67 [b]	16.30 [a]	19.57 [a]	15.48 [b]	16.03	18.45	1.33	<0.01	0.15	<0.01
Lactate[4]														
D-lactate, mM	0.186	0.195	0.640	0.650	0.633	0.723	0.872	0.893	0.699	0.795	0.0015	<0.01	0.25	<0.05
L-lactate, mM	0.123	0.099	0.313	0.310	0.249	0.289	0.290	0.406	0.348	0.390	0.0007	<0.01	0.27	<0.01
Total lactate, mM	0.330	0.311	0.972	0.967	0.863	1.020	1.194	1.309	1.102	1.219	0.0020	<0.01	0.27	<0.01

[1] CON: A control diet containing no phytogenic product; PHY: supplementation with a phytogenic feed additive based on L-menthol, thymol, eugenol, mint oil (*Mentha arvensis*) and cloves powder (*Syzygium aromaticum*); [2]The largest standard error of the mean; [3]*p*-Values for the effect of diet (D), phytogenic treatment (T) and the diet × week × treatment interaction (I); [4]Values were transformed using the root square function after checking for normal distribution, and were transformed back after the analysis; [a,b] Means with different superscripts indicate a significant difference ($p < 0.05$) between CON and PHY; [x,y] Means with different superscripts indicate a tendency for significant difference ($0.05 < p \leq 0.10$) between CON and PHY.

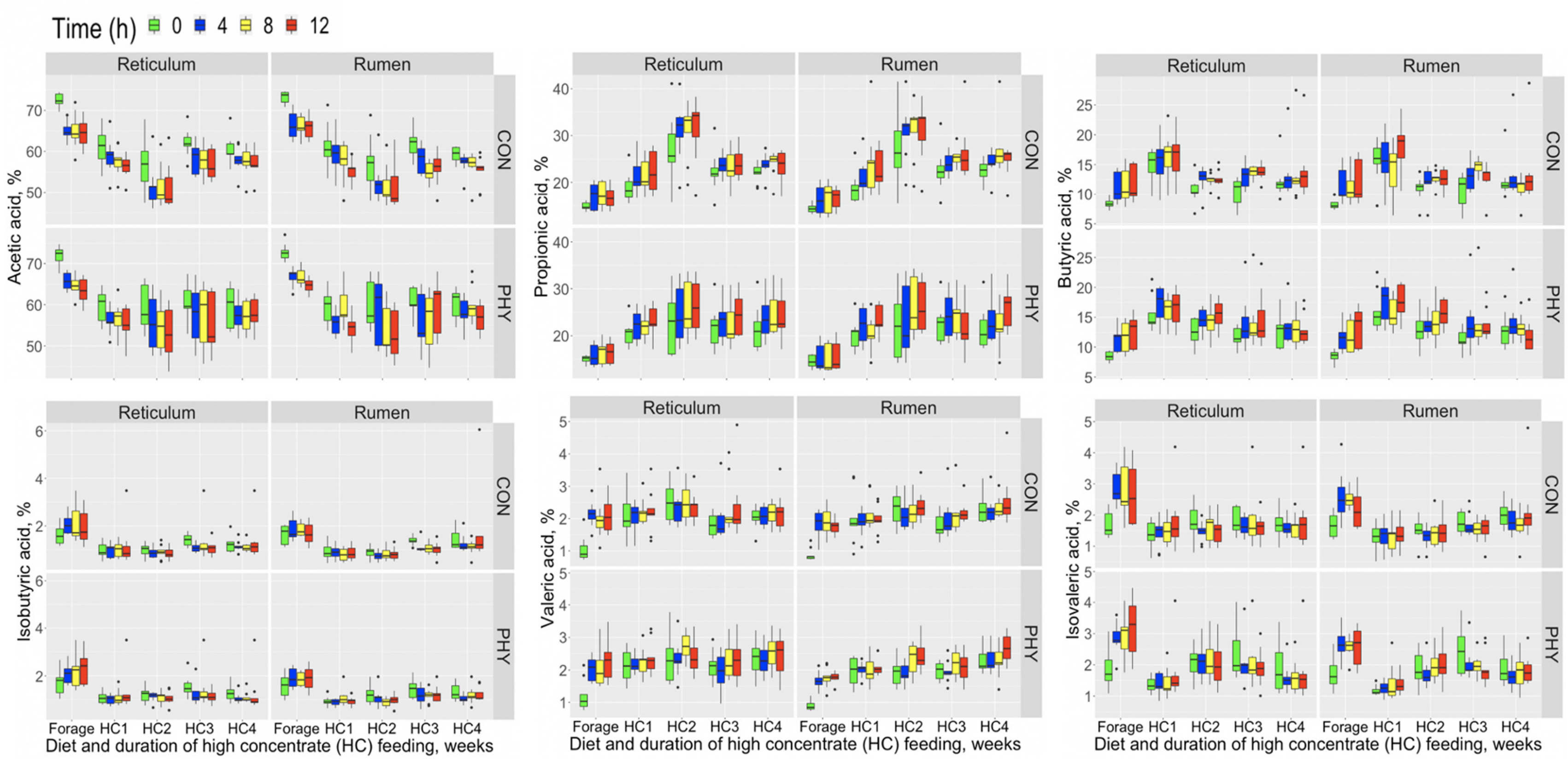

Figure S1. Boxplots illustrating the fermentation pattern and short chain fatty acid profile in the reticulum and rumen with time post-feeding according to diet and duration of high concentrate feeding in Holstein cows without supplementation (CON) or supplemented with a phytogenic feed additive (PHY) based on L-menthol, thymol, eugenol, mint oil (*Mentha arvensis*) and cloves powder (*Syzygium aromaticum*).

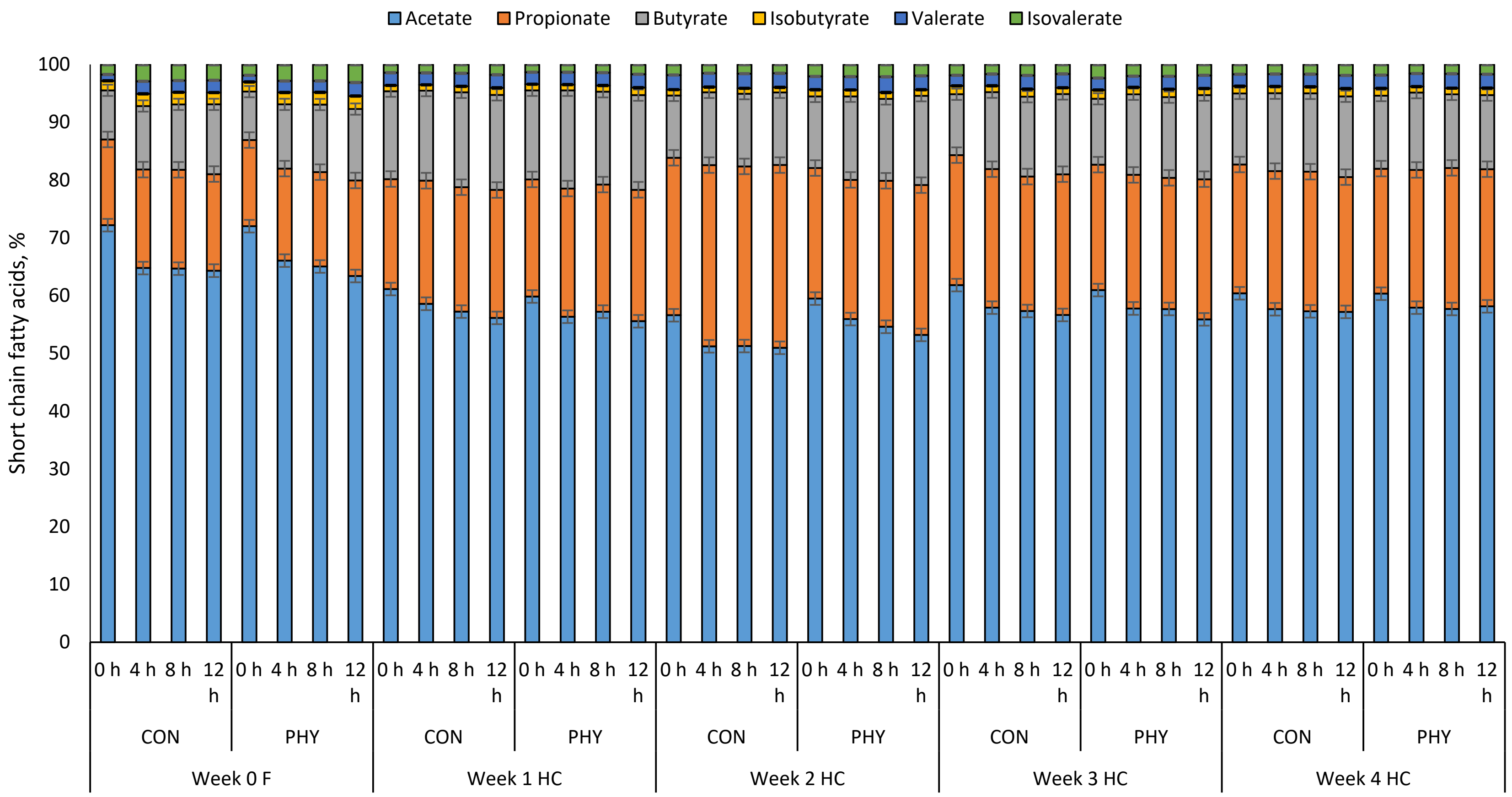

Figure S2. Variation of reticular short chain fatty acid fermentation from 0 to 12 h post-feeding in cows fed either all-forage (F) or a high concentrate (HC), without supplementation (CON) or supplemented with a phytogenic feed additive (PHY) based on L-menthol, thymol, eugenol, mint oil (*Mentha arvensis*) and cloves powder (*Syzygium aromaticum*). *p*-Values: Acetate, Time < 0.01, Trt $= 0.54$, Diet < 0.01, Time×Trt×Diet×Week < 0.01; Propionate, Time < 0.01, Trt $= 0.17$, Diet < 0.01, Time×Trt×Diet×Week < 0.01; Butyrate, Time < 0.01, Trt $= 0.50$, Diet < 0.01, Time×Trt×Diet×Week < 0.01; Isobutyrate, Time $= 0.42$, Trt $= 0.67$, Diet < 0.01, Time×Trt×Diet×Week < 0.01; Valerate, Time < 0.01, Trt $= 0.64$, Diet < 0.01, Time×Trt×Diet×Week < 0.01; Isovalerate, Time < 0.05, Trt $= 0.29$, Diet < 0.01, Time×Trt×Diet×Week < 0.01.

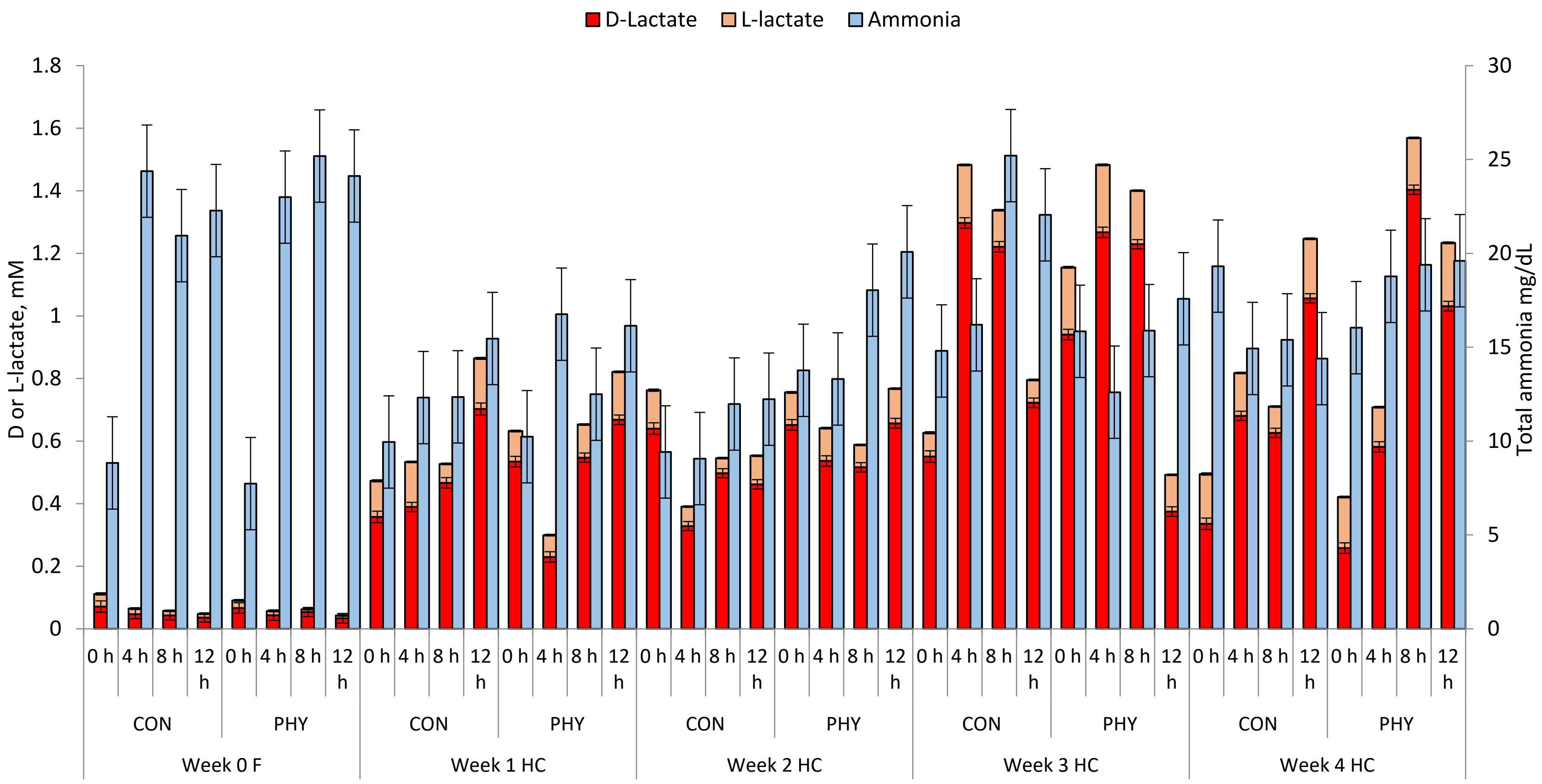

Figure S3. Variation of reticular D-lactate, L-lactate (mM), and total ammonia concentration (mg/dL) from 0 to 12 h post-feeding in cows fed either all-forage (F) or a high concentrate (HC), without supplementation (CON) or supplemented with a phytogenic feed additive (PHY) based on L-menthol, thymol, eugenol, mint oil (*Mentha arvensis*) and cloves powder (*Syzygium aromaticum*). *p*-Values: D-lactate, Time = 0.25, Trt = 0.48, Diet < 0.01, Time×Trt×Diet×Week < 0.01; L-lactate, Time = 0.16, Trt = 0.38, Diet < 0.01, Time×Trt×Diet×Week < 0.05; Total ammonia, Time < 0.01, Trt = 0.15, Diet < 0.01, Time×Trt×Diet×Week < 0.01.
